# Tumour ploidy and prognosis in carcinomas of the bladder and prostate.

**DOI:** 10.1038/bjc.1966.53

**Published:** 1966-09

**Authors:** A. S. Tavares, J. Costa, A. de Carvalho, M. Reis


### TUMOUR PLOIDY AND PROGNOSIS IN CARCINOMAS OF THE BLADDER AND PROSTATE

A. S. TAVARES, J. COSTA, A. DE CARVALHO AND M. REIS

*From the Departments of General Pathology and of Urology, University of Oporto Faculty of Medicine, and the Department of Urology, Hospital Escolar de S. João, Oporto, Portugal*

Received for publication March 14, 1966

It is very difficult to establish the prognosis for a patient bearing a malignant tumour, and every physician has had his own share of errors, due to lack of objective criteria, besides the type and size of the growth, and the existence of metastases and/or local invasion.

The recent development of cytogenetical techniques enabled Atkin (1964) to introduce new criteria for prognosis in carcinoma of the cervix, criteria which are based on the cellular ploidy as assessed by DNA measurements, and Tavares *et al.* (1964) have shown a difference in the metastasizing capacity of diploid and tetraploid cell lines of female breast carcinomas.

In this paper evidence of a correlation between ploidy and survival after surgery of patients with malignant epithelial growths of the bladder and prostate is reported.

### MATERIAL AND METHODS

A series of 62 cases of transitional cell carcinoma* of the bladder and 35 of adenocarcinoma of the prostate, operated upon by three of us and examined at the Department of Morbid Anatomy, University of Oporto Faculty of Medicine (Prof. A. Tavares), was selected on the basis of adequate follow-up. Papillomas of the bladder, which may be considered as grade 0 carcinomas, were not included here and will be studied independently. Patients were followed during variable periods of time after surgery ; roentgen or isotope therapy was used in 39 cases of the bladder series, and oestrogen therapy in 33 of the prostate carcinomas.

Paraffin sections of the material for histological examination obtained at the time of operation were treated by Feulgen's method for DNA, after 30 minutes hydrolysis in 1 N HCl, at 60° C. The optical density of 30 nuclei, chosen at random in carcinomatous areas, was determined in each slide using a Deeley integrating microdensitometer (model G.N.2, Messrs. Barr and Stroud, Ltd., Glasgow), at 5600 Å, and compared with that of 5 to 10 neutrophils in the same areas. DNA nuclear contents were recorded in L units, an L unit being defined as 1·1 times the average density of neutrophils (Atkin, 1962), and the data plotted in a frequency histogram, with a logarithmic scale in ordinates.

Evaluation of ploidy through the examination of these histograms may appear somewhat subjective in some cases (specially when large number of cells in S period occur), so a statistic, $k_0$, was calculated according to Maia (1966) : assuming a bimodal distribution of DNA values in a homogeneous cellular population,

* Tumour types as defined by U.I.C.C. ' Illustrated Tumor Nomenclature ', Springer-Verlag.

$k_0$ corresponds to the theoretical DNA value of null dispersion around the primary mode (Tavares and Maia, 1966), and is easily determined by the formula

$$k_0 = \frac{3\Sigma X \pm \sqrt{9\Sigma^2 X - 8n\Sigma X^2}}{4n}$$

where $n$ is the number of nuclei read in one slide and $X$ the individual DNA value per nucleus. Histograms with highly dispersed values may yield imaginary $k_0$, and a subjective evaluation of ploidy is then necessary but, in general, $k_0$ ranges are 1–1·4 in diploid lines (2C), 1·5–2 in triploid lines (3C), 2–2·8 tetraploid lines (4C), and 3–4 in hexaploid lines (6C). However, values near the mentioned limits are still not discriminatory enough, and a way to overcome this is being investigated, with the help of other parameters ; meanwhile, examination of histograms must be resorted to in these cases, specially when there are a number of elements outside the context of a well defined modal distribution.

### RESULTS

### *Carcinoma of the bladder*

From the 62 cases, all of them operated upon using the same technique (transurethral resection), 35 were classified as diploid or tetraploid, and included in the group 2–4C : 13 of the patients (37%) died between 1 and 9 years after surgery (average : 33·6 months), two of them from causes apparently unrelated to their bladder growth. In the triploid-hexaploid group (3–6C), 27 tumours were counted, with a higher mortality rate, 21 of the patients dying between 1 month and 6 years after the operation (average : 18·1 months). Differences in the response of both groups to roentgen or isotope therapy are not clear cut, and a larger series is needed to ascertain a correlation between ploidy and sensitivity to irradiation.

Actuarial methods permitted the comparison of global survival rates in either group with that calculated by Maia (1955) for the Portuguese male population (Fig. 1) : median expectation of life for Portuguese males aged 62 (average age in the bladder series) is 13·5 years, and the median life expectation after surgery is 6·2 and 1·6 years, respectively in 2–4C and 3–6C groups. In this series, the fact of a patient having been operated upon for a bladder carcinoma means a reduction of 55% in his median life expectation if the tumour is diploid or tetraploid, and 88% if it is triploid or hexaploid.

### *Carcinoma of the prostate*

As regards prostatic carcinomas, from 26 patients with 2–4C growths, 7 died after a mean survival period of 7·4 years, while in the 3–6C group 7 of the 9 patients died after 4·1 years in average.

The number of cases (35) does not allow a reliable actuarial analysis for the 3–6C group ; it was done nevertheless, since there is a suggestion of high difference in prognosis between the two groups (Fig. 2). Median life expectation for the Portuguese male population aged 66 (average age of the patients in the prostate series) is 10·3 years ; there is a slight drop, to 9·4 years (91%) in the 2–4C group, and a greater one, to 4·2 years (41%) in the 3–6C group.

Also interesting, and undoubtedly related to prognosis, is the difference between the groups as regards the response to oestrogen therapy, in the 33 patients

FIG. 1.—Carcinoma of the bladder : survival rates after transurethral resection in the two groups defined by tumours ploidy (2–4C and 3–6C), as compared with the survival rate computed for Portuguese males aged 62. Broken lines indicate the points of medium life expectation.

FIG. 2.—Carcinoma of the prostate : comparison of survival rates, after surgery, of patients with 2–4C and 3–6C tumours, and the survival rate computed for Portuguese males aged 66. Broken lines indicate the points of median life expectation.

who were submitted to it (Table I) : all but one of 9 3–6C carcinomas were found resistant to oestrogens (in one case, a triploid growth was only partially and temporarily reduced by therapy), while 22 of 24 2–4C tumours responded well.

### COMMENTS

The different behaviour of malignant neoplasms of the bladder and of the prostate may be related to chromosomal complement differences—as is assumed for cancer in general. However, definition of the karyotype is usually difficult

TABLE I.—*Carcinomas of the Prostate: Ploidy, Prognosis and Response to Oestrogens*

| | Group 2–4C | Group 3–6C |
|---|---|---|
| Prognosis | | |
| Number of patients alive . | 19 ($s = 3{\cdot}3$) | 2 ($s = 1$) |
| Number of patients dead . | 6 ($s = 6{\cdot}4$) | 7 ($s = 2{\cdot}9$) |
| Response to oestrogens (33 cases) | | |
| Good . . . . . . | 22 | 1 |
| None . . . . . . | 2 | 8* |

$s$ : average survival after surgery, in years.
* includes one case of partial resistance (see text).

and cannot be routinely done, hence the resort to DNA measurements as a means to define the ploidy of stem-line cells. Paraffin sections, as used in this investigation, do not allow high precision in individual determinations, but present the advantage of permitting the revision of large series of cases which have been filed away for a long time. This is a good reason for their use, as material obtained with more refined cytological techniques is as yet limited.

It is not easy to discuss at this moment the causes for the different behaviour in both groups. It may be suggested that maintained or altered gene equilibrium (in 2–4C and 3–6C strains, respectively) will probably intervene as a factor in the development of a given tumour. There is suggestive evidence, in the different response to prostate carcinomas to oestrogens, of a metabolic influence of an imbalance due to trisomy (3C) or double-trisomy (6C), but the results obtained in this series, as those reported by Atkin (1964) for cervix carcinomas, should not be generalized to all sites: it appears that ploidy may control differently the prognosis, depending on the type and localization of the tumour, so that such a correlation should be investigated individually for each organ. It may be interesting to note here that a similar situation exists for breast carcinomas, where sex chromatin may be regarded as a criterion for hormonal treatment (Hienz and Ehlers (1957); see review in Tavares, 1966).

This investigation was done under a grant from the Portuguese Institute for Higher Culture to the Research Centre for Morbid Anatomy and General Pathology (Faculty of Medicine, University of Oporto).

### REFERENCES

ATKIN, N. B.—(1964) *Cancer, N.Y.*, **17**, 1391.—(1962) *Cytogenetics*, **1**, 113.
HIENZ, H. A. AND EHLERS, P. N.—(1957) *Klin. Wschr.*, **35**, 985.
MAIA, J. C.—(1966) *Médico, Porto* (in press).—(1955) *Médico, Porto*, **3**, 583.
TAVARES, A. S.—(1966) 'Sex chromatin in tumours' in 'Sex chromatin', edited by Moore, K. L. New York (Saunders), Chapter 23.
TAVARES, A. S. AND MAIA, J. C.—(1966) *Médico, Porto* (in press).
TAVARES, A. S., MAIA, J. C. AND PEREIRA, J. M. R.—(1964) *Médico, Porto*, **32**, 6.


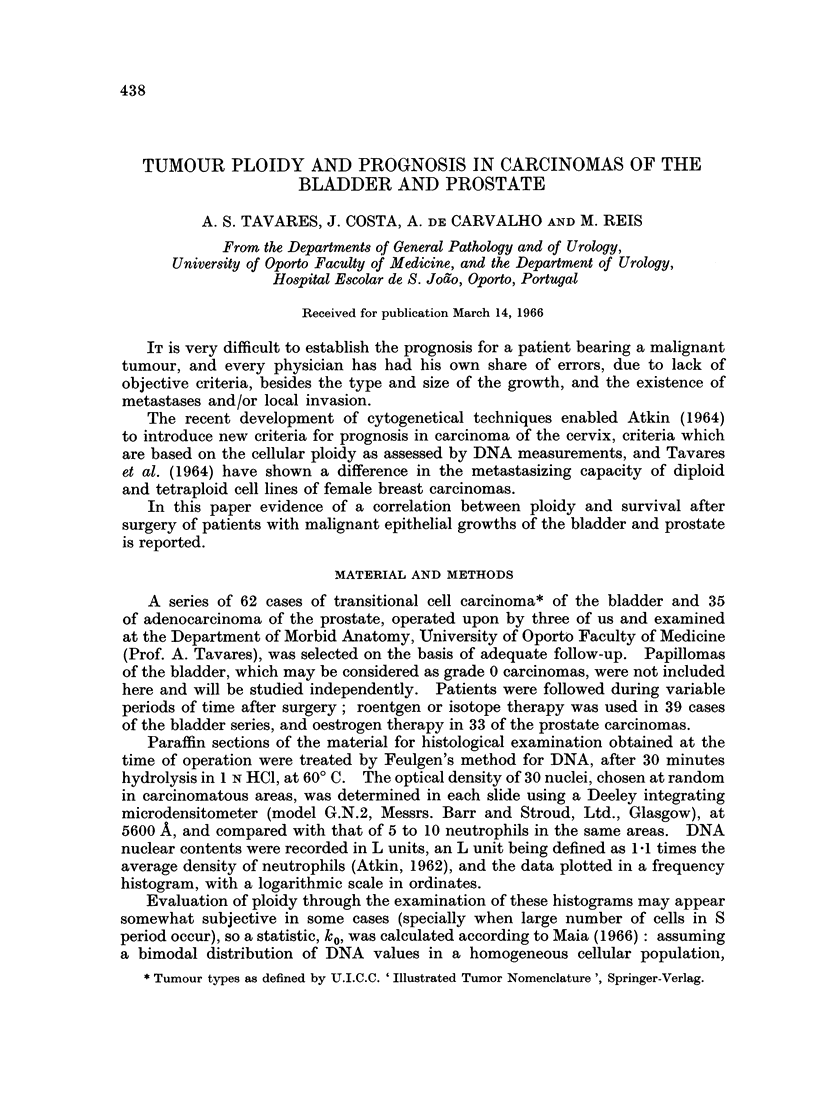

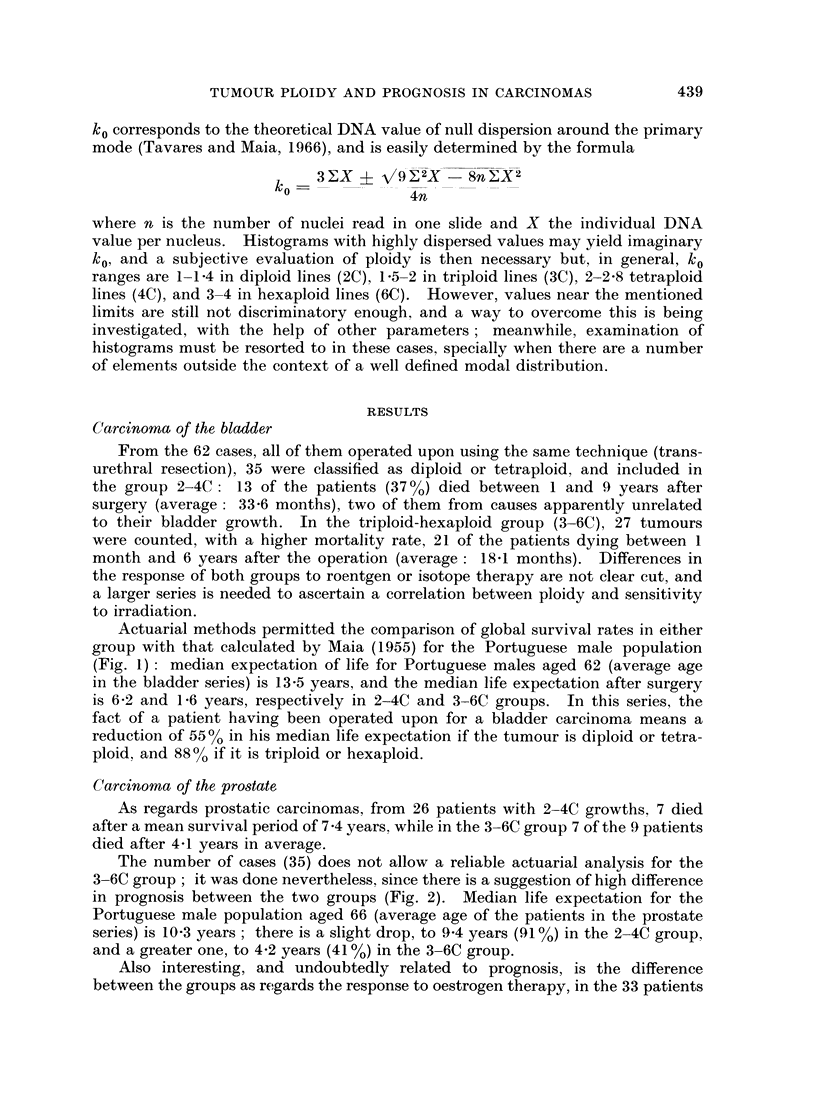

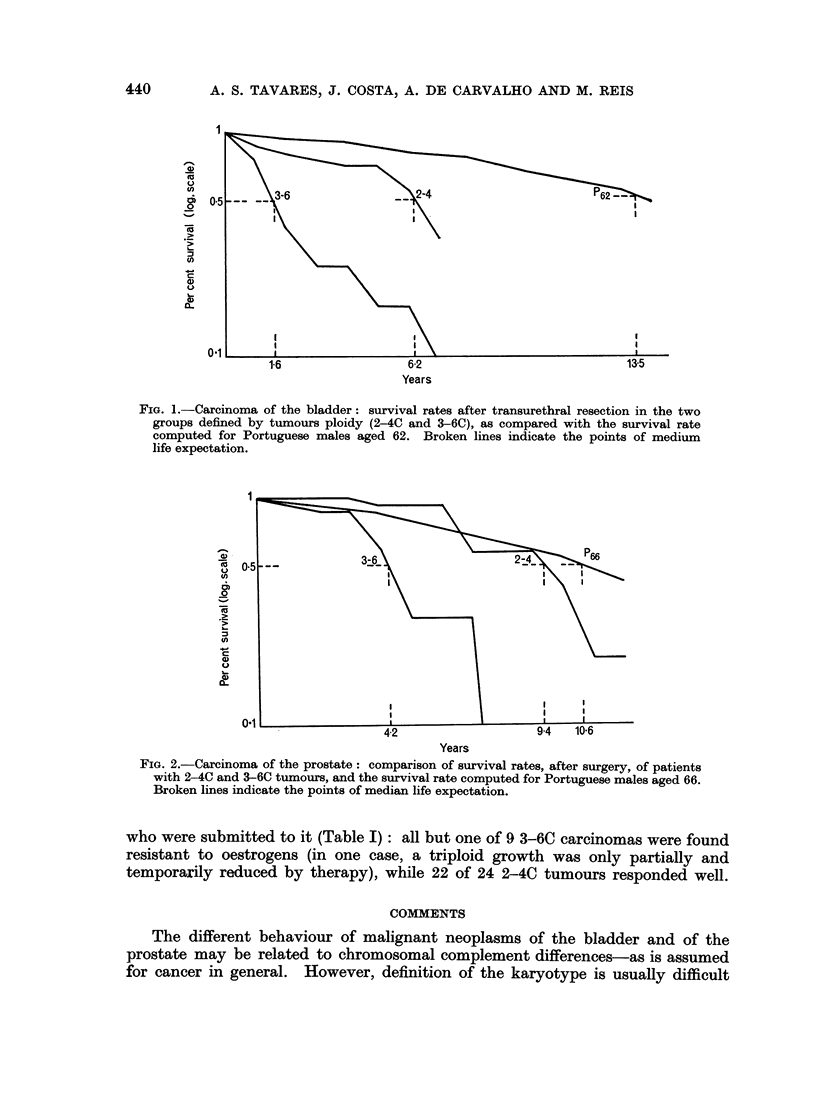

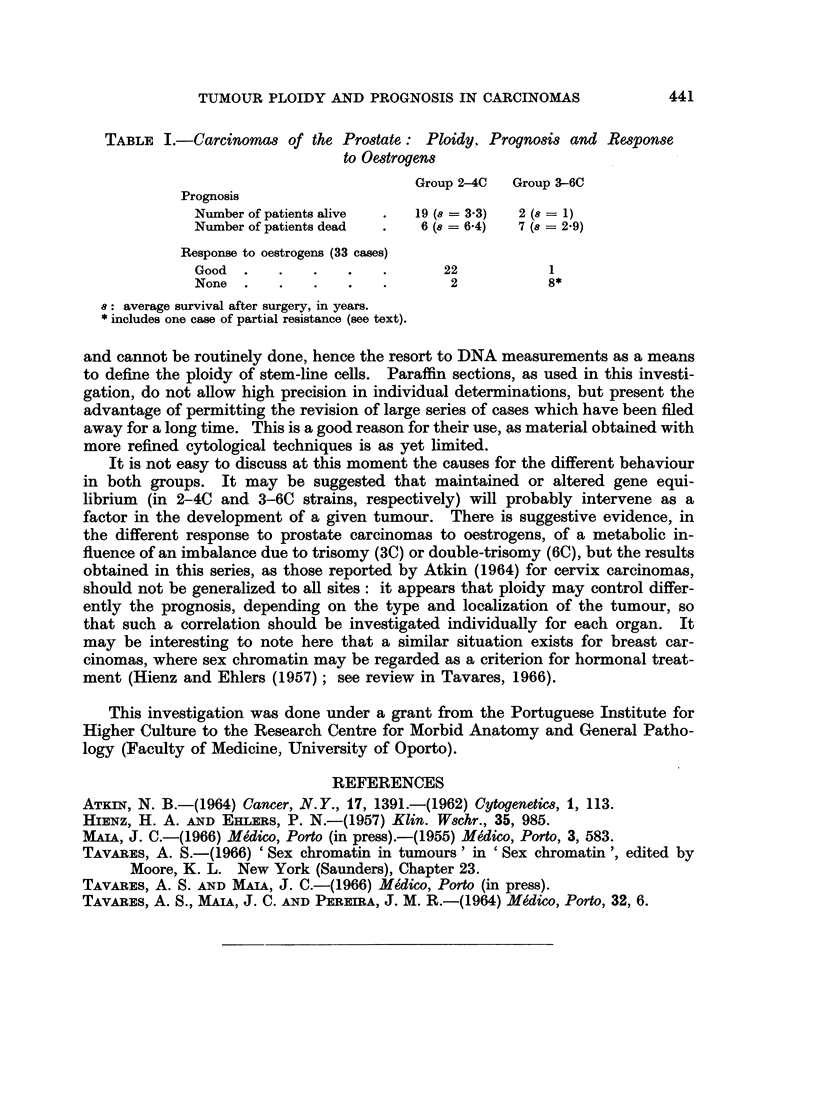


## References

[OCR_00219] HIENZ H. A., EHLERS P. N. (1957). Das zellkernmorphologische Geschlecht von Mamma- und Prostatacarcinomen.. Klin Wochenschr.

